# Bioactives in Chinese Proprietary Medicine Modulates 5α-Reductase Activity and Gene Expression Associated with Androgenetic Alopecia

**DOI:** 10.3389/fphar.2017.00194

**Published:** 2017-04-13

**Authors:** Justin J. Y. Tan, Jing Pan, Lihan Sun, Junying Zhang, Chunyong Wu, Lifeng Kang

**Affiliations:** ^1^Department of Pharmacy, National University of SingaporeSingapore, Singapore; ^2^Department of Pharmaceutical Analysis, China Pharmaceutical UniversityNanjing, China; ^3^Key Laboratory of Drug Quality Control and Pharmacovigilance, Ministry of Education, China Pharmaceutical UniversityNanjing, China; ^4^Department of Pharmaceutics of Traditional Chinese Medicine, China Pharmaceutical UniversityNanjing, China

**Keywords:** Yangxue Shengfa capsule, 5α-reductase, androgenetic alopecia, dermal papilla, HPLC

## Abstract

Androgenetic alopecia (AGA) is characterized by a progressive and patterned transformation of thick, pigmented terminal scalp hairs into short, hypo-pigmented vellus-like hairs. The use of Minoxidil and Finasteride to treat AGA are often associated with complications in safety and efficacy. However, herbal remedies are deemed to have lesser side effects in many societies. This study aims to identify potential hair growth properties of individual compounds from a Chinese proprietary medicine known as Yangxue Shengfa capsule (YSC), used in China for many years for improving AGA. Six marker compounds, including 2,3,5,4'-tetrahydroxystilbene-2-O-β-D-glucoside (TSG), Chlorogenic acid, Emodin, Ferulic acid, Isoimperatorin, and Paeoniflorin were used for simultaneous HPLC quantification and anti-AGA *in-vitro* screening. Simultaneous quantification of these components was performed on 75% (v/v) methanol extracts of YSC, using a Welch Ultimate XB-C18 column and gradient elution. Five compounds significantly promoted cell proliferation in cultured immortalized human Dermal Papilla Cells (DPC). Multiple genes associated with the progression of AGA, including IGF-1, DKK-1, and TGF-β1, were found to be regulated by some of these compounds. Interestingly, Ferulic acid and Emodin demonstrated good pharmacological properties against AGA, thereby concluding the potential of these bioactives to be used in the treatment against AGA.

## Introduction

Androgenetic alopecia (AGA), also commonly known as male pattern hair loss in men and female pattern hair loss in women, is generally characterized by a progressive and patterned transformation of thick, pigmented terminal scalp hairs into short, fine, hypo-pigmented vellus-like hairs (Kaufman, 2002). The link between androgens and alopecia was first established in a paper published by (Hamilton, 1942; Kaufman et al., 1998). In his work, it was observed that castrated males did not exhibit any observable signs of hair loss unless challenged with doses of testosterone in individuals with genetic predisposition toward AGA. However, a discontinuation of testosterone administration reverses the hair loss in these males. Their low levels of circulating testosterone and its enzymatic product, Dihydrotestosterone (DHT or 5α-DHT), catalyzed by the 5α-reductase enzyme (Kaufman et al., 1998), were therefore espoused for their possible association to the preservation of terminal scalp hairs. DHT demonstrated about five-fold greater affinity for the androgen receptor and 10-fold higher potency for causing hair loss as compared to its precursor substrate, testosterone (Jain et al., 2015). Strategies to overcome AGA include blocking the activity of 5α-reductase with medication such as finasteride, which is a selective inhibitor of type II isoform of 5α-reductase (Ahmed and Denison, 1998) and competitively blocks the conversion of testosterone to 5α-DHT in the prostate, retarding the progression of Benign Prostatic Hyperplasia in males (Weisser et al., 1994). Another approved drug for AGA is Minoxidil, which was previously used as an anti-hypertensive medication. Minoxidil acts via the opening of the adenosine triphosphate sensitive potassium channel to induce vascular smooth muscle relaxation, accounting for its hypotensive effects (Andersson, 1992). This mechanism by which it acts to induce hypertrichosis in sufferers of AGA remains poorly understood. It was however, postulated that Minoxidil was able to upregulate production of growth factors in cultured Dermal Papilla Cells (DPC) which might have attributed to the hair growth (Lachgar et al., 1998).

The use of Minoxidil and Finasteride in treating AGA are often associated with complications in safety and efficacy. Finasteride has been reported to alter systemic steroid metabolism, leading to conditions such as metabolic syndrome, insulin resistance, and diabetes (Traish et al., 2014). Both Minoxidil and Finasteride require lengthy durations of treatment before they can achieve considerable effectiveness (Falto-Aizpurua et al., 2014) and the chances of relapses upon discontinuation is high (Guerouaz and Mohamed, 2014). These inconsistencies in drug efficacy and adverse drug effects relating to their use have led to the search for more potent and safer agents to treat AGA. Herbal remedies are deemed to have lesser side effects than western medications in many societies (Yoon et al., 2010), with numerous undocumented cases of efficacious use of these processed herbs. However, this may mask an underlying biasness as many herbal products are known to interact with drug metabolizing enzymes, thus altering drug-plasma levels and originating toxic effects in western medicines (Mancuso and Barone, 2009; Mancuso et al., 2012; Mancuso, 2015). In addition, concentrations used in *in-vitro* studies to obtain pharmacological effects are, very often, much higher than those effectively reaching the blood and tissues, alluding to their poor bioavailability, though strategies such as complexation with non-toxic carriers help to improve their absorption and distribution (Mancuso, 2015). Having said this, composition elucidation in drug discovery becomes a crucial process for identifying bioactives and understanding their unique pharmacodynamic and pharmacokinetic properties helps to further drug development. The growing research interests in pharmacognosy and significant discoveries stemming from natural products—such as the anti-malarial drug Artemisinin and the anti-cancer drug, vincristine, derived from *Catharanthus roseus* (Madagascar periwinkle), have paved the way for many modern drug discoveries. Similarly, screening was done on many natural products for activity against AGA. Among these include *Panax ginseng* (Kim et al., 2015), *Polygonum multiflorum* (Sun et al., 2013), and *Chamaecyparis obtuse* (Lee et al., 2010).

The Chinese proprietary medicine we used in this study is known as Yangxue Shengfa capsule (YSC), an officially listed formulation in the Chinese Pharmacopeia (Pharmacopoeia of the People's Republic of China, 2015) and has been used in China for many years for the improvement of multiple conditions involving hair loss, including alopecia areata, AGA and postpartum alopecia. It is a capsule formulation composed of nine different herbal ingredients, including *Radix Rehmanniae praeparata, Radix Angelicae sinensis, Rhizoma et Radix Notopterygii, Fructus Chaenomelis, Rhizoma Chuanxiong, Radix Paeoniae alba, Semen Cuscutae, Rhizoma Gastrodiae, Radix Polygoni Multiflori praeparata*. However, the complexity of its chemical composition has limited the elucidation of its efficacy mechanism against AGA and hair loss. Based on our previous work, six marker compounds including 2,3,5,4'-tetrahydroxystilbene-2-O-β-D-glucoside (TSG), Chlorogenic acid, Emodin, Ferulic acid, Isoimperatorin, and Paeoniflorin have been identified in the YSC by HPLC-DAD-MS (data not shown). The objective of this present research aims to simultaneously quantify these six marker constituents in YSC and subsequently screen them for potential activity against AGA. We hypothesized that the marker constituents found in YSC can incite hair growth through DPC proliferation, 5α-reductase inhibition, and modulation of genes associated to AGA pathogenesis.

## Results

### Optimization of the preparation and chromatographic conditions for the analysis of YSC

To achieve an efficient extraction of multiple components in YSC, different extraction methods (hot reflux or ultrasonication), extracting solvents (0, 50, 75, and 100% methanol or alcohol, v/v), extraction time (0.5, 1, or 2 h) and drug to solvent ratios (1:10, 1:25, 1:50, and 1:75, m/v), were investigated. Considering the balance between extraction efficacy and detection sensitivity, the optimized extraction conditions were identified as follows: 75% (v/v) methanol as solvent; hot reflux for 30 min with a drug to solvent ratio of 1:50.

**Figure 1:**
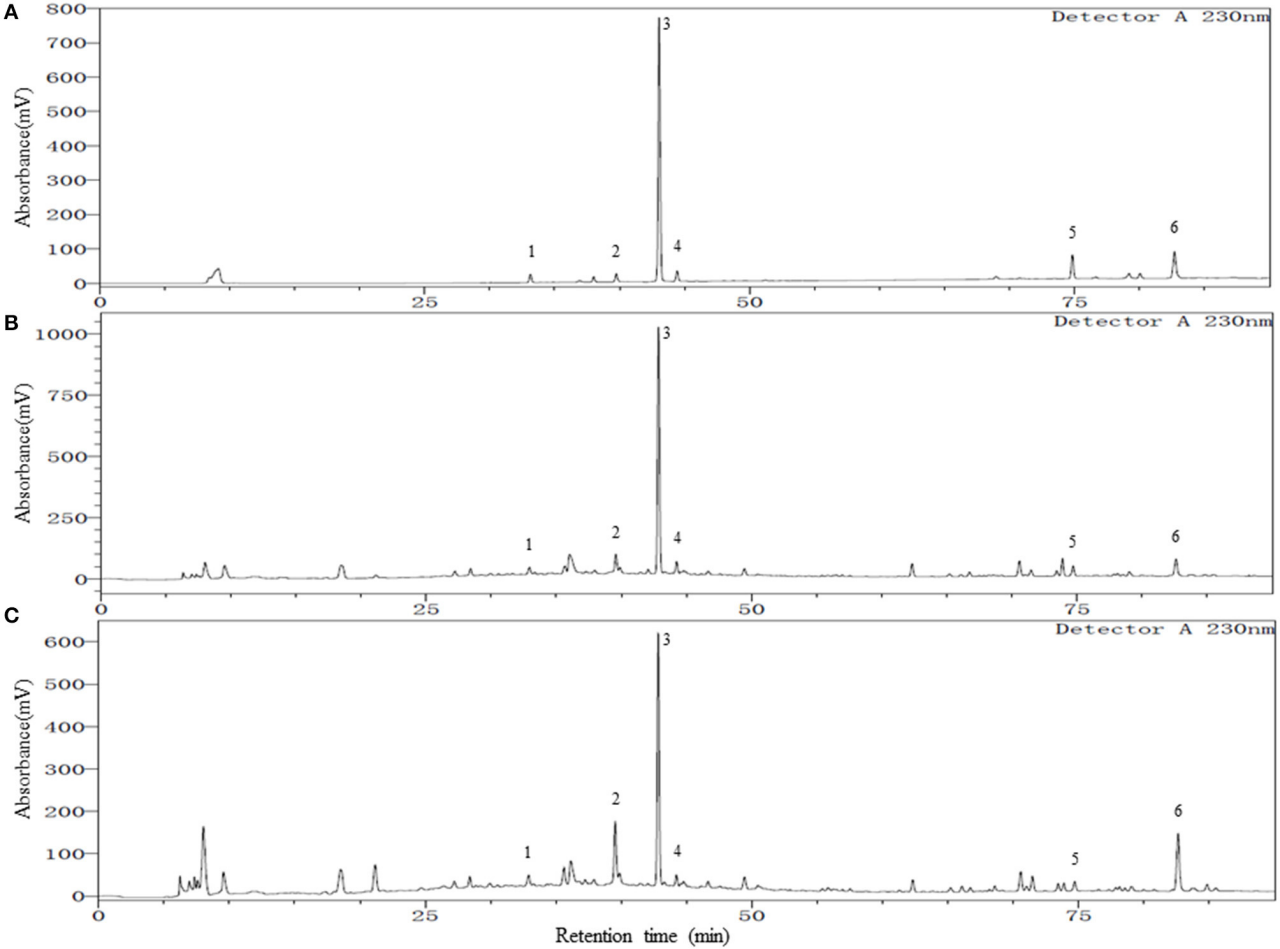
**Representative chromatograms of mixed reference standards (A)**, YSC samples: Lot E01104B **(B)**, and Lot Y03039 **(C)**. Peak numbers represent the following compounds: 1. Chlorogenic acid; 2. Paeoniflorin; 3. 2,3,5,4′-tetrahydroxylstilbene-2-O-β-D-glucoside (TSG); 4. Ferulic acid; 5. Isoimperatorin; 6. Emodin.

To achieve a satisfactory separation of the multiple components in YSC, different high performance liquid chromatography (HPLC) parameters, including the mobile phases, columns, columns temperature, and detection wavelengths were examined. The column temperature of 30°C and a wavelength of 230 nm provided good separation and satisfactory UV absorption for all investigated compounds. The mobile phase was then developed by investigating different composition, pH, and gradient elution profiles. Since methanol and acetonitrile presented similar separation ability, methanol-water system was selected due to their low cost. The pH of the mobile phase was found to have a great influence on the peak shape, retention times, and resolution of the constituents of YSC. Therefore, different acids (acetic acid, formic acid, phosphoric acid) and pH-values (2–4.5) were evaluated, and best resolution as well as baseline were obtained at pH 3.0 by adding phosphoric acid into the water phase. An optimum gradient elution mode described in the Methods and Materials section was used for better chromatographic separation on a wide range of polarity in shorter time. Finally, different C18 columns were compared, and a Welch Ultimate XB-C18 column (4.6 × 250 mm, 3 μm) was employed. The representative chromatograms of a mixed standard solution and YSC samples are shown in Figure 1. The retention factors of chlorogenic acid, paeoniflorin, TSG, ferulic acid, isoimperatorin and emodin were 4.3, 5.4, 5.9, 6.1, 11.0, and 12.2, respectively, indicating adequate migration rate of each analyte under the developed HPLC conditions.

### Quantitative analysis of the six marker compounds in YSC

The optimized HPLC method was validated with respect to linearity and range, limit of quantification (LOQ), precision, repeatability and accuracy, and the results are shown in Table 1.

**Table 1:** **Method validation for the simultaneous quantification of six marker compounds in YSC**.

**Compound**	**Regression equation**	**Linear range (μg/ml)**	**LOQ (ng/ml)**	**Precision (RSD%)**	**Repeatability (RSD%)**	**Recovery**	
						**Mean/%**	**RSD/%**
TSG	y = 43458x − 3800 (*r*^2^ = 0.9994)	17.0–510.0	38	0.69	0.34	100.4	0.90
Chlorogenic acid	y = 40829x − 7401 (*r*^2^ = 0.9993)	0.8–24.0	40	0.78	1.70	98.23	1.42
Emodin	y = 110508x +16873 (*r*^2^ = 0.9993)	1.1–33.0	35	0.69	2.03	97.45	1.64
Ferulic acid	y = 85449x + 19.7 (*r*^2^ = 0.9992)	0.4–12.0	40	0.80	3.07	97.28	2.71
Isoimperatorin	y = 95469x − 14901 (*r*^2^ = 0.9991)	0.9–27.0	18	0.82	0.88	101.7	2.35
Paeoniflorin	y = 36849x − 1256 (*r*^2^ = 0.9996)	1.6–48.0	50	0.76	0.74	96.91	1.87

As shown in Table 1, all the six compounds showed good linearity (*r*^2^ > 0.9991). The LOQ varied from 18 to 50 ng/ml. The precision and repeatability (RSD %) were found to be not more than 0.82 and 3.07% for all analytes, respectively. In the present study the components of YSC were extracted by reflux for 30 min in 75% methanol. Although the extraction of ferulic acid may be sensitive to such procedures which caused its repeatability slightly worse than other compounds, the overall repeatability is acceptable for the herbal medicine analysis. The six analytes showed good recovery of 96.91–101.7%. Therefore, the method is sensitive, precise and accurate, and has been successfully applied for the simultaneous analysis of chlorogenic acid, paeoniflorin, TSG, ferulic acid, isoimperatorin, and emodin in two brands of YSC, and the results are shown in Figure 1 and Table 2.

**Table 2:** **Contents (mg/g) of six marker compounds in two brands of YSC (***n*** = 3)**.

**Compound**	**Contents (mg/g) (mean** ± ***SD*****)**	
	**Lot E01104**	**Lot Y03039**
TSG	12.23 ± 0.05	7.25 ± 0.06
Chlorogenic acid	0.48 ± 0.01	0.46 ± 0.01
Emodin	0.49 ± 0.01	0.94 ± 0.02
Ferulic acid	0.32 ± 0.01	0.17 ± 0.01
Isoimperatorin	0.29 ± 0.01	0.17 ± 0.01
Paeoniflorin	1.36 ± 0.01	2.68 ± 0.02

In both YSC samples, TSG and paeoniflorin were detected as the dominant constituents. Although the contents of the six marker compounds except chlorogenic acid showed variation, all these six compounds were detected in both brands of YSC, indicating that they may be the active components of YSC and they were selected as the marker compounds for the *in-vitro* anti-AGA screening.

### DPC proliferation assay

**Figure 2:**
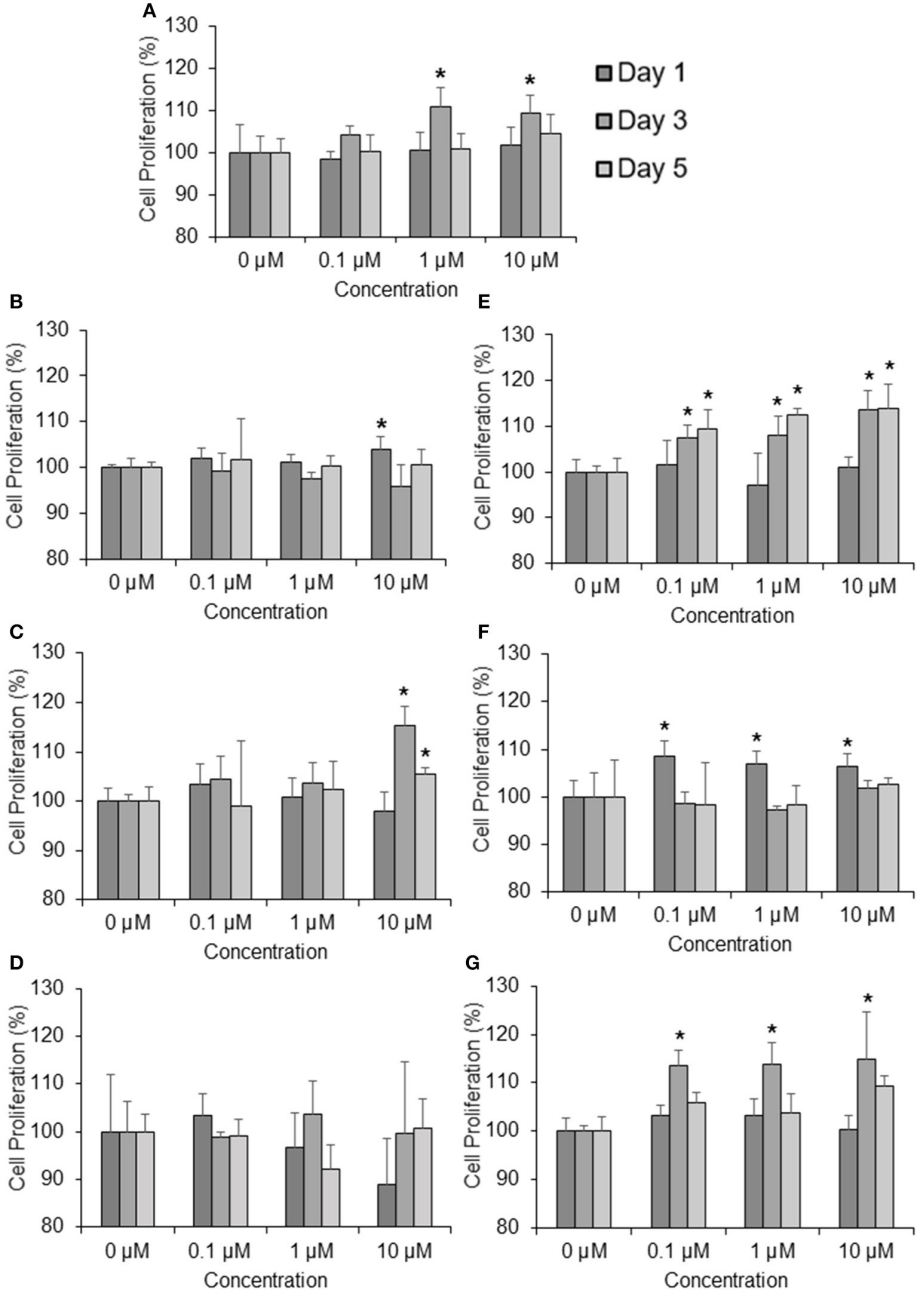
**Compounds found from the YSC induce human DPC proliferation**. Concentrations tested are 0.1, 1, 10 μM at 1, 3, 5 days. **(A)** Minoxidil, **(B)** 2,3,5,4 tetrahydroxystilbene-2-O-β-D-glucoside (TSG), **(C)** Chlorogenic Acid, **(D)** Emodin, **(E)** Ferulic Acid, **(F)** Isoimperatorin, **(G)** Paeoniflorin ^*^P < 0.05 vs. 0 μM.

To determine the effects of the six marker compounds on DPC proliferation, MTT-Thiazolyl blue [3-(4,5-dimethylthiazol-2-yl)-2,5-diphenyl tetrazolium bromide]—assay was performed on DPC with the treatment of seven compounds—minoxidil (Positive control), TSG, Chlorogenic Acid, Emodin, Ferulic Acid, Isoimperatorin, and Paeoniflorin for three concentrations—0.1, 1, and 10 μM at three incubation time points—1, 3, and 5 days. Significant proliferation (*p* < 0.05) in DPC were mainly observed in all of the compounds assayed except Emodin (Figure 2D). Among these compounds, TSG (Figure 2B) and chlorogenic acid (Figure 2C) only showed activity at the highest concentration, i.e., 10 μM tested, while ferulic acid (Figure 2E), isoimperatorin (Figure 2F), and paeoniflorin (Figure 2G) were shown to be effective at all the concentrations tested. However, minoxidil (Figure 2A), the positive control used in this assay, were observed to be effective at concentrations 1 and 10 μM. When compared across the incubation time points, TSG and Isoimperatorin were only able to induce significant DPC proliferation at the day 1, while Minoxidil and Paeoniflorin were only able to induce significant DPC proliferation at the day 3. Ferulic acid and chlorogenic acid were able to induce significant DPC proliferation on day 3–5 of incubation.

### 5α-reductase inhibition assay

**Figure 3:**
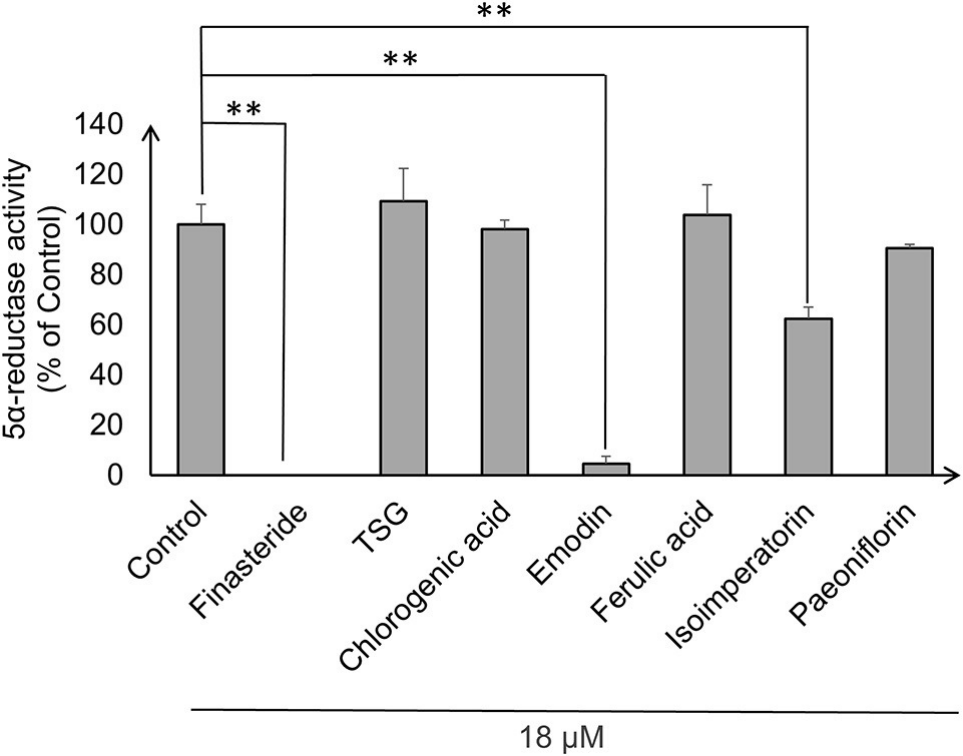
**5α-reductase activity was measured in DPC using LC-MS/MS**. 5α-reductase activity was calculated using the ratio of DHT produced after drug treatment to DHT produced in control expressed as a percentage of control. ^**^P < 0.01 vs. control.

Androgens, especially DHT, were known to be highly implicated in hair regression and balding in genetically predisposed individuals. Thus, we decided to investigate whether the compounds have any effects on 5α-reductase activity in the DPC. To this end, we performed an *in-vitro* cell based 5α-reductase assay based on a previously established method (Jain et al., 2015). Prior to the analysis of the samples, a standard calibration plot was done with seven concentrations of DHT using Mesterolone as the internal standard. DHT showed good linearity over the concentration range of 50–1,000 ng/ml with correlation coefficient of *r*^2^ ≥ 0.99. The typical regression equation of DHT was y = 0.0067x + 0.1227, where y indicates the ratio of sample to control signal peak area. Among the seven compounds screened, three compounds showed significant 5α-reductase inhibition activity (*p* < 0.01; Figure 3). Levels of DHT were undetectable in the Finasteride (Positive control) treated group, while emodin and isoimperatorin showed significant reduction in DHT levels with the emodin-treated group and the isoimperatorin-treated group showing 4.5 and 62.3% of DHT produced at the end of the 48 h incubation, respectively.

### Quantitative real-time PCR analysis

Real-time polymerase chain reaction (RT-PCR) was subsequently performed to determine the expression of six genes affecting the health of hair follicles and their progression in AGA. In this study, we measured the expression levels of 5a-reductase II (SRD5A2), Androgen receptor (AR), β-catenin, Dickkopf-1 (DKK-1), Insulin-like Growth Factor-1 (IGF-1), Transforming Growth Factor-beta 1 (TGF-β1), and Glyceraldehyde-3-phosphate dehydrogenase (GAPDH) as the housekeeping gene. The gene expression levels were normalized to the housekeeping gene (GAPDH) and expressed as a percentage of the non-treated group (vehicle only control group).

**Figure 4:**
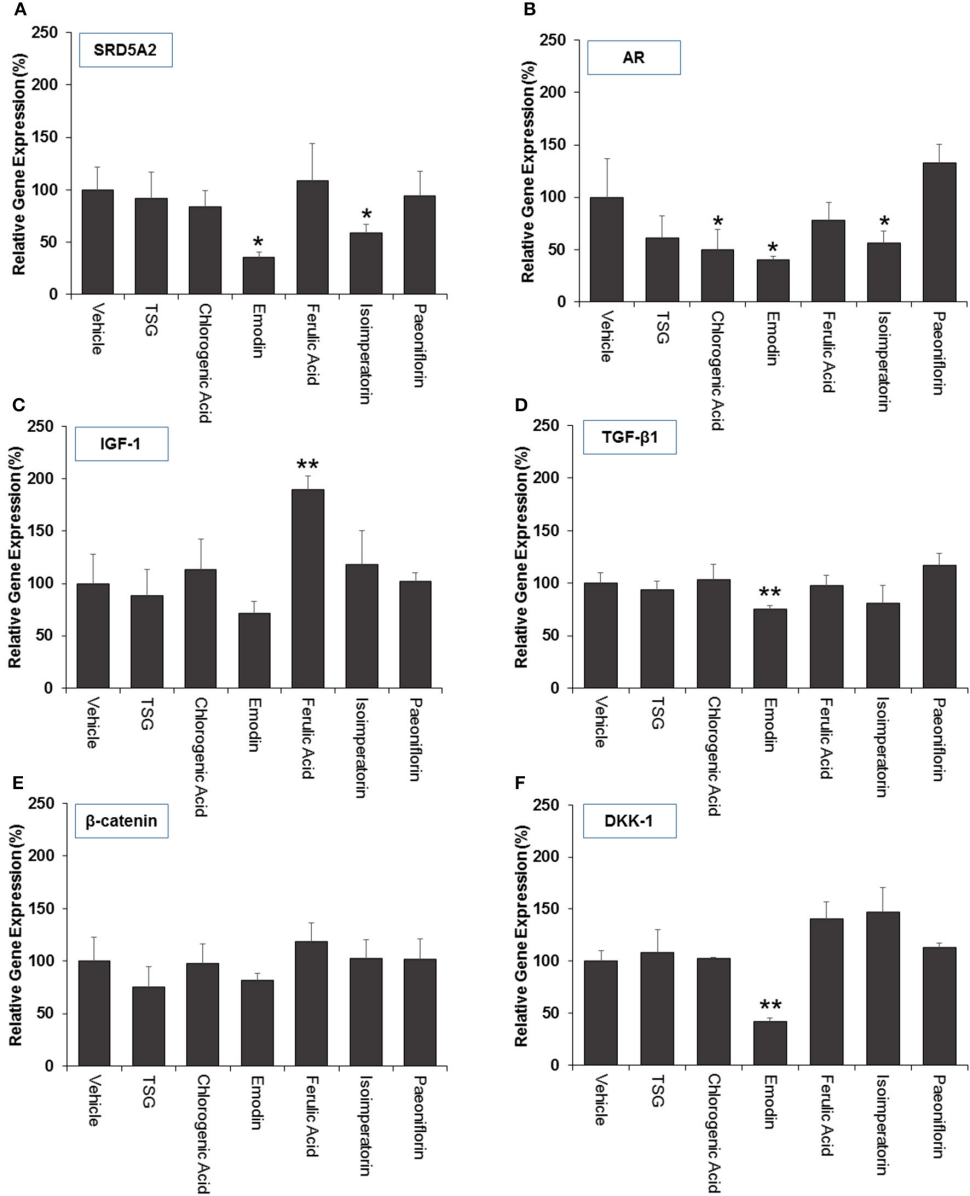
**The effect marker compounds on (A)** 5α-reductase II **(B)** androgen receptor **(C)** IGF-1 **(D)** TGF-β1 **(E)** β-catenin and **(F)** DKK-1 gene expression in DPCs. Cells were treated with each compound for 72 h and gene expression analyzed using real-time PCR. The level of gene expression in the non-treated (vehicle) group was set to 100%. The graphs summarized the experimental analyses (mean ± *SD*). ^*^P < 0.05 vs. vehicle group ^**^P < 0.01 vs. vehicle group

For selected genes that were involved in the androgen signaling pathway, SRD5A2 was downregulated significantly (*p* < 0.05) by emodin and isoimperatorin (Figure 4A), by 64.5 and 41.0%, respectively while the expression of AR was significantly reduced (*p* < 0.05) by chlorogenic acid, emodin, and isoimperatorin (Figure 4B) by 49.7, 59.5, and 43.9%, respectively.

Selected key growth factor genes that play essential roles in hair cycle regulation, primarily IGF-1 and TGF-β1 also showed significant changes (*p* < 0.01) in expression levels after treatment with ferulic acid and emodin. IGF-1 showed an 89.9% increase in expression level after treated with Ferulic Acid (Figure 4C) while TGF-β1 showed a 24.7% decrease in expression level after treated with Emodin (Figure 4D).

For selected genes involved in the Wnt signaling pathway, primarily β-catenin and DKK-1, there were no significant changes to the levels of the β-catenin gene expression (Figure 4E) while Emodin significantly downregulated (*p* < 0.05) the expression of DKK-1 gene expression by 58.2% (Figure 4F).

## Discussion

Current *in-vitro* screening of compounds for AGA treatment were modeled after the proposed mechanisms of minoxidil and finasteride. Hence, “ideal therapeutics” for AGA often requires a combination of 5a-reductase inhibitors and hair growth promoters (Randall, 2008). The direct influence of minoxidil on the proliferation of DPC has allowed it to serve as a model for subsequent drug screening (Han et al., 2004). The sizes of the human dermal papillae, which depended on the number of DPC, have been correlated with hair growth, and the number of DPC increases during the growth phase of the hair cycle (Park et al., 2015). Miniaturized hairs or vellus-like hairs, a cardinal sign observed in many cases of AGA, was believed to be caused by the depletion of DPC number, forcing the hair follicle to experience a premature anagen phase (growth phase), shortening it from an average of 2–7 years to just several months (Kaufman, 2002). As a result, many studies used DPC proliferation as a preliminary assessment for DPC growth inducers and a model for evaluating their potential as anti-AGA agents (Han et al., 2004; Park et al., 2015). In our study, DPC proliferation was measured using the MTT assay to determine the number of viable cells remaining in the culture at three time points—day 1, 3, and 5 after incubation with the compounds at concentrations 0.1, 1, and 10 μM. The concentrations tested were within the working concentrations of Minoxidil used in previous literature known to work on DPC (Han et al., 2004). The assessment of DPC proliferation across multiple time-points and different compound concentrations allows for greater understanding of the onset and the potency of the compounds inducing DPC proliferation. Among the compounds which could induce significant proliferation (*p* < 0.05) in DPC, ferulic acid was shown to be the most potent agent, effectively inducing DPC proliferation at concentrations as low as 0.1 μM and maintaining significant proliferation over the course of 3 days, from day 3 to 5. Other compounds in general, showed modest, non-sustained proliferation on either day 1 or 3, with the exception of chlorogenic acid, which caused sustained proliferation in the DPC from day 3 to 5 at 10 μM. Minoxidil, the positive control used in this study, was only able to induce significant proliferation in DPC at concentrations of 1 μM and higher, although minoxidil was reported to be able to induce greater proliferation in DPC at concentrations as low as 0.01 μM (Han et al., 2004). The relatively slow onset of DPC proliferation on day 3 after minoxidil treatment may explain the refractory period associated with minoxidil use before any signs of terminal hair growth can be observed. Normal DPC proliferation resumed on day 5 post-minoxidil treatment, suggesting the need for regular use of minoxidil to maintain the high DPC numbers and hence, prolonging the anagen phase of the hair cycle in AGA afflicted hair follicles. Therefore, Ferulic Acid has shown to be more effective than minoxidil in initiating DPC proliferation at a lower concentration and sustaining DPC proliferation over a longer duration. The depletion of DPC in AGA-afflicted hair follicles have been attributed to the influence of androgens (mainly Testosterone and DHT), mainly by apoptosis (Winiarska et al., 2006). Other mechanisms of androgen-mediated hair follicle miniaturization in AGA extends the apoptotic influence of androgens to cells surrounding the DPC, such as the outer root sheath keratinocytes (Kwack et al., 2008). Mainstream therapeutics such as finasteride acts to inhibit the enzyme 5α-reductase to lower the plasma levels of DHT circulating in the bloodstream (Lee and Lee, 2012). To this end, the effects of the compounds on 5α-reductase activity in the DPC were investigated. An effectively high concentration of 18 μM for each compound was tested to assess any potential 5α-reductase inhibition activity within the DPC. In our study, compounds emodin and isoimperatorin were shown to possess activity against 5α-reductase in DPC, especially with Emodin in reducing the overall production of DHT. The influence of the compounds on the expression of molecules mediating the molecular signaling pathway of androgens in DPC was studied with RT-PCR. Gene expression was studied on DPC after 72 h incubation with the compounds of interest at 10 μM to identify the pattern of sustained gene expression over this sufficiently long duration. Treatments for hair growth are often protracted, and sustained gene expression is a crucial feature for achieving this purpose. A 3-day or 72 h incubation would be viable to ensure that sufficiently “long time” is given to determine the efficacy of the drug in the DPC. Second, DPC would be able to retain their cellular integrity without overcrowded conditions within the culture. Thirdly, most compounds in the DPC proliferation assay shown favorable increase on day 3 or 72 h, hence, studying the gene expression allows for possible correlation between gene expression and cellular proliferation. The gene expression of 5α-reductase II (SRD5A2), the predominant enzyme expressed in abundance within DPC (Inui and Itami, 2011), and androgen receptor (AR) was evaluated in DPC treated with the compounds. It was observed that emodin and isoimperatorin, the two compounds which exhibited 5α-reductase inhibitory activity, also significantly downregulated the expression of SRD5A2 and AR, with emodin showing a greater effect as compared to Isoimperatorin. A similar trend was observed with 5α-reductase inhibition in DPC. Androgens, especially DHT, is known to be implicated in the Wnt-signaling pathway. The Wnts belong to a family of secreted glycoproteins which acts on responding cells by activating one or more of several signal transduction pathways within the cells (Fotaki et al., 2009). The β-catenin signal transduction cascade, activated by the Wnts, plays essential roles in hair follicle formation and regeneration (DasGupta and Fuchs, 1999; Huelsken et al., 2001; Andl et al., 2002; DasGupta et al., 2002; Maretto et al., 2003; Van Mater, 2003). The association was established in a study by Kwack et al, which identified Dickkopf-1 (DKK-1) gene as one of the most upregulated DHT-inducible genes in DPC (Kwack et al., 2008). DKK-1 is a potent and specific endogenously secreted Wnt antagonist which binds and inhibits low-density lipoprotein receptor-related protein co-receptors required for canonical Wnt signaling involved in hair induction and growth (Chiang et al., 1999; Millar et al., 1999; Kishimoto et al., 2000; Shimizu and Morgan, 2004). In addition, DKK-1 was observed to be more highly expressed in balding scalp as compared to healthy scalp lysates (Kwack et al., 2008). Kwack et al. have also demonstrated that DHT significantly reduced the growth of outer root sheath (ORS) cells in co-cultured with DPC and neutralizing anti-DKK-1 antibody significantly attenuated the DHT-induced growth inhibition in ORS cells (Kwack et al., 2008). In our findings, we observed that emodin is able significantly downregulate the production of DKK-1 by more than 50% of control, and hence, may potentially be able to act as an agent against DHT-induced growth inhibition in ORS of AGA-afflicted patients. Androgens have also reported to exert influence over the expression of key genes essential for the regulation of the human hair cycle, namely at the anagen (growth phase) and the catagen (regression phase) of the human hair cycle. In our study, we have chosen to evaluate the expression of two important genes regulating these key phases of the human hair cycle, namely Insulin-like Growth Factor 1(IGF-1) and Transforming Growth Factor beta 1(TGF-β1). IGF-1 is one of the most extensively studied gene for its essential role in hair cycle control as well as hair shaft differentiation during the development of hair follicles. In its absence, anagen hair follicles in organ culture enter the catagen phase (Panchaprateep and Asawanonda, 2014). DPC from balding scalp follicles were found to secrete significantly less IGF-1 as compared to non-balding scalp follicles (Panchaprateep and Asawanonda, 2014). This phenomenon was alluded to the prolongation effect of the anagen phase by IGF-1. Human hair follicles treated with exogenous IGF-1 had their anagen stage prolonged, and the average length of the hair shaft showed greater elongation than non-treated controls (Ahn et al., 2012). The effects of IGF-1 were also evaluated in animal models, especially mice. Local injection of IGF-1 increased the growth of cultured hair follicles and facilitated the migration of hair in developing hair follicles in transgenic mice (Li et al., 2014). IGF-1 receptor knockout mice showed significant reduction in the number of hair follicles, abnormal hair follicular pattern, and hair differentiation (Panchaprateep and Asawanonda, 2014). The mitogenic effect of IGF-1 was observed in our study when ferulic acid was incubated with DPC over the course of 5 days and showed sustained proliferation from day 3 to 5. It was also the single agent among the compounds tested to have significantly upregulated the production of IGF-1 in DPC by close to 200% of control. The upregulation of IGF-1 by Ferulic acid may be a promising sign that Ferulic acid may potentially be used as an agent to restore the IGF-1 levels in AGA patients. TGF-β1, on the other hand, is commonly referred to as the catagen inducer in the human hair cycle because it triggers early onset catagen in AGA affected hair follicles, preventing the development of the hair shaft into thick, pigmented terminal hairs (Inui and Itami, 2011). DPC from balding scalp secreted TGF-β1 in response to androgens, and this inhibited the growth of the surrounding epithelial cells (Inui et al., 2002). Although the mechanism of how TGF-β1 resulted in catagen remains unclear, it was proposed that catagen is inherently an apoptotic process and TGF-β proteins are able to generate caspases leading to cellular apoptosis (Hibino and Nishiyama, 2004). In our study, all our compounds tested did not exhibit any upregulation of TGF-β1 expression, while Emodin was able to significantly downregulate its expression by about 25% of the control. Table 3 summarizes the results of all the *in-vitro* assays performed to demonstrate the individual compound's potential in the treatment against AGA.

**Table 3:** **Summary table demonstrating individual compound's potential in the treatment of AGA**.

**Compound**	**DPC Proliferation**	**5α-reductase inhibition**	**Gene expression**					
			**SRD5A2**	**AR**	**IGF-1**	**TGF-β1**	**β-catenin**	**DKK-1**
TSG	√	–	–	–	–	–	–	–
Chlorogenic acid	√	–	–	↓	–	–	–	–
Emodin	–	√	↓	↓	-	↓	–	↓
Ferulic acid	√	–	–	–	↑	–	–	–
Isoimperatorin	√	√	↓	↓	–	–	–	–
Paeoniflorin	√	–	–	–	–	–	–	–

## Conclusion

*In-vitro* screening models are predictive models aiding our understanding and discovery of potential compounds for the treatment of AGA. However, the complexity of a Chinese proprietary medicine may render the screening of individual drug component implausible. In our study, we have successfully developed a HPLC method to simultaneously identify six marker compounds from YSC, namely TSG, chlorogenic acid, emodin, ferulic acid, isoimperatorin, and paeoniflorin, which will help to improve the quality control of YSC. These marker constituents are subsequently screened for potential activities in DPC proliferation, 5α-reductase inhibition, and regulation of genes essential for the progression of AGA in DPC. We have identified two promising compounds which may effectively be able to slow the progression of AGA in human DPC, namely Ferulic acid and emodin. Ferulic acid showed good proliferative properties in DPC and upregulated IGF-1, an essential growth factor for prolonging the anagen (growth) phase in the hair cycle. Emodin exhibited good inhibitory activity against 5α-reductase and significantly lowered the levels of DHT in DPC, while also reducing the expression of two most upregulated genes in AGA—TGF-β1 and DKK-1. Our findings, therefore allows the identification of bioactives against AGA to be used in future study and research.

## Methods and materials

### Materials

Reference compounds, including chlorogenic acid, paeoniflorin, Ferulic acid, 2,3,5,4'-tetrahydroxystilbene-2-O-β-D-glucoside (TSG), isoimperatorin, and emodin with their purities all over 98.0% were purchased from Nanjing Spring & Autumn Biological Engineering Co. Ltd. (Nanjing, China). Dihydrotestosterone, testosterone, finasteride and minoxidil (≥ 98% purity) were purchased from Sigma-Aldrich (St. Louis, USA). Methanol was HPLC grade and purchased from Tedia (Fairfield, USA). The Chinese proprietary medicine capsules YSC were produced by Two pharmaceutical companies (Lot E01104 and Y03039, respectively). The ultra-pure water from a Milli-Q system (Millipore) was used for all analyses. Immortalized human DPC were kindly donated by Professor Mike Philpott and Dr Adiam Bahta from Queen Mary University London (QMUL). Dulbecco's modified Eagle's medium (DMEM), fetal bovine serum (FBS), random primers and SYBR safe DNA gel stain were supplied by Invitrogen, Life Technologies (USA). Trypsin and penicillin/streptomycin solution were obtained from PAN-Biotech GmbH (Germany). 3-(4,5-dimethylthiazol-2-yl)-2,5-diphenyl tetrazolium bromide (MTT) and dimethyl sulfoxide (DMSO) were acquired from MP Biomedicals (Illkirch, France). RNeasy Mini Kit and QuantiFast SYBR Green PCR kit were purchased from Qiagen (Germany). Random primers and avian myeloblastosis virus reverse transcriptase were purchased from Promega (Madison, Wisconsin, USA). All other reagents were of analytical grade obtained from conventional commercial sources and used as supplied.

### Simultaneous quantification of multiple marker components in YSC

#### Preparation of reference and test solutions

Stock solutions of the six reference substances (Chlorogenic acid, Paeoniflorin, TSG, Ferulic acid, Isoimperatorin, and Emodin) were accurately weighed and dissolved separately with the concentration ranging from 25 to 100 mg/mL in DMSO, followed by dilution with methanol to make a solution of 1 mg/ml. They were stored at 4°C until use. The working solutions of standard mixture of six standards was prepared by diluting stock solutions in 75% (v/v) methanol to desired concentrations.

For sample preparation, mix thoroughly the contents of 20 capsules, weigh accurately 1.5 g in a stopper conical flask, add accurately 75 ml of 75% (v/v) methanol and weigh. Heat under reflux for 30 min, stand to cool, replenish the lost weight with 75% (v/v) methanol, and mix well. Filter through a 0.45 μm membrane filter, and use the successive filtrate as the test solution.

#### Instrumentation and conditions

HPLC analysis was performed on a Shimadzu LC-2010A equipped with a quaternary pump, vacuum degasser, autosampler, column oven, and UV detector (Shimadzu, Kyoto, Japan). LabSolutions software was used for instrument control, data acquisition and procession. The separation was carried out on a Welch Ultimate XB-C18 column (4.6 × 250 mm, 3 μm) with the column temperature at 30°C, and the flow rate was 0.4 ml/min. The mobile phase consisted of phosphoric acid solution (pH 3.0) (A) and methanol (B). The following gradient elution program was used: 0–8 min, 10–15% B; 8–35 min, 15–49% B; 35–66 min, 49–85% B; 66–76 min, 85–90% B; 76–85 min, 90% B; 85–95 min, 10% B. Analytes were monitored at 230 nm. The injection volume was 10 μL.

#### Method validation

The stock solutions of six reference compounds were diluted to provide five-point calibration levels, and the calibration curves were established by plotting the peak area vs. concentration of each analyte. The contents of six marker compounds in YSC samples were calculated from the corresponding calibration curve. The limit of quantification (LOQ) for each standard were estimated at a signal-to-noise ratio (S/N) of not <10. Working solution of the middle concentration level was injected six times successively for the precision test. Six replicates of the same sample (Lot E01104) were extracted and analyzed to investigate the repeatability. Recovery was investigated by spiking the YSC sample (Lot E01104) with reference standards at three levels, followed by the extraction and analysis, and three replicates were performed for each level. Variations were expressed in terms of relative standard deviation (RSD).

### Cell culture

The immortalized dermal papilla cell line was donated by Prof. Mike Philpott and Dr. Adiam Bahta from Queen Mary University London (QMUL) for this work. The cell lines were previously isolated and immortalized from dermal papilla obtained from scalp biopsy (Chew et al., 2016).

The dermal papilla cell line (p30–33) were used for all the *in-vitro* experiments in this paper. The cell lines were manipulated under aseptic conditions and maintained in a humidified incubator at 37°C and 10% CO_2_. Culture media consisted of DMEM (Invitrogen Corporation, USA) supplemented with 10% FBS and 1% penicillin-streptomycin (10,000 U/ml penicillin and 10 mg/ml streptomycin, PAN-Biotech GmbH, Germany). All media components were sterilized via filtration through 0.22 μm pore Corning filter units (Corning Incorporated, USA). Culture medium was changed every 3 days and cells were passaged when flasks were 80–90% confluent.

### Cell proliferation assay

To determine the effect of the various marker compounds of YSC on DPC survival and proliferation, viability was measured using the MTT assay. DPC were seeded at a density of 2,000 cells in 200 μl of culture medium into each well of 96-well plates. After 24 h, the existing media was aspirated and replaced with 200 μl of fresh media, together with the indicated concentrations of each test compound and incubated for 1, 3, and 5 days. 0.05% (v/v) methanol or DMSO was used as negative control. Then, the medium was replaced with an equal volume of fresh medium and 20 μL of MTT (1 mg/ml) in phosphate buffer saline (PBS) was added. At the end of 4 h incubation, medium containing the untransformed MTT was removed. One hundred microliters of DMSO was subsequently added to each well and the plates were incubated for 10 min and shaken for about 2 min. The optical density (OD) at 570 nm (reference wavelength: 660 nm) was determined using a Tecan plate reader (Infinite 200 PRO, Switzerland). The cell viability rates were calculated from the OD readings and are represented as percentages of the control value (untreated cells) for 0.05% (v/v) methanol or DMSO, according to solvent which was used in the sample preparation.

### 5α-reductase inhibition assay

#### Drug treatment and sample preparation

DPC were seeded at a cell density of 1 × 10^5^ cells/ml onto 96-well plates (100 μl of 10,000 cells/well). After 24 h, the cells were separately treated (in a total volume of 200 μl) with the final concentrations of testosterone (18 μg/ml), test compounds (18 μM) and <1% Methanol or 1% DMSO as the negative control. After 48 h, the culture medium of each treatment was collected in Eppendorf tubes, and the attached cells were lysed using 200 μL of 0.1 N sodium hydroxide (NaOH) in each well. The cell lysates were transferred into the respective Eppendorf tubes (containing the culture medium) and extracted with the 1 mL of ethyl acetate twice with a brief vortex to ensure homogeneity between each extraction for 30 s. The ethyl acetate layer was then evaporated to dryness and reconstituted using 100 μL of methanol. The internal standard (IS) chosen for this assay was mesterolone. Samples were then injected into LC-MS/MS for subsequent analysis.

#### LC-MS/MS analysis

Liquid chromatography was performed on a Agilent 1200 series liquid chromatography (Germany). The separation was carried on a Agilent ZORBAX Eclipse Plus C18 column (3.0 × 150 mm, 5 μm) protected with a Zorbax-SB C18 guard column maintained at room temperature. The mobile phase composition was 75% of methanol (with 0.1% formic acid) and 25% of water (with 0.1% formic acid) in isocratic mode at a flow rate of 0.5 ml/min. The injection volume was 10 μl. An Agilent 6400 series triple quadrupole mass spectrometer equipped with Turbo Ion Spray interface operating in the positive electrospray ionization (ESI) mode was used for the detection. Operating conditions optimized by Mass Hunter optimizer for DHT and IS were: dry gas temperature 350°C; nebulizer pressure 35 psi; nitrogen gas flow rate 10 ml/min; capillary voltage 4,000 V and fragmentor voltage 130 V for DHT and 140 V for IS. Product ions (of DHT) resulting from transition of 291.2 → 255.2 (collision energy 12 eV) and product ions (of IS) 305.2 → 93.1 (collision energy 36 eV) were monitored at retention time of 5.5 min (for DHT) and 6.3 min (for IS).

### mRNA extraction

mRNA was extracted from cells using the RNeasy Mini Kit in accordance with the manufacturer's instructions. The concentration of mRNA was determined using NanoDrop 1000 Spectrophotometer (Thermo Scientific, USA). Reverse transcription of total mRNA was performed at 1 μg of total mRNA in 25 μl final volume using random primers and avian myeloblastosis virus reverse transcriptase. The concentration of complementary deoxyribonucleic acid (cDNA) after reverse transcription were also determined using NanoDrop Spectrophotometer.

### Quantitative real-time polymerase chain reaction (PCR)

Quantitative Real-Time PCR reaction was performed using Rotor-Gene Q Real-Time PCR cycler (Qiagen, Germany). Primers (Integrated DNA technologies, Singapore) used for PCR reactions were listed in Table 4.

**Table 4:** **DNA sequence of primer pairs used for quantitative real time PCR**.

**Gene symbol**	**Gene name**	**Forward primer (5′–3′)**	**Reverse primer (5′–3′)**
β-Catenin	β-Catenin	AAGCAGAGATGGCCCAGAAT	AGTGGGATGGTGGGTGTAAG
GAPDH	Glyceraldehyde 3-phosphate dehydrogenase	TGAAGGTCGGAGTCAACGG	TGGAAGATGGTGATGGGAT
Androgen Receptor	Androgen Receptor	GGGACCATGTTTTGCCCATT	GCAGCTTCCACATGTGAGAG
DKK-1	Dickkopf-1	CCATTGACAACTACCAGCCG	CTGCAGGCGAGACAGATTTG
IGF-1	Insulin-like Growth Factor-1	CATGTCCTCCTCGCATCTCT	TGTCTCCACACACGAACTGA
TGF-β1	Transforming Growth Factor-beta 1	AGACTTTTCCCCAGACCTCG	TGGGTGGTCTTGAATAGGGG
5α-reductase II	5α-reductase II	GCTTCATACCCACTCCCTGT	TGGGTCTTTGTGGCTTCAGA

Primer sequences were designed using Primer3 (http://frodo.wi.mit.edu/) and Primer-BLAST (http://www.ncbi.nlm.nih.gov/tools/primer-blast/). Each reaction mixture was prepared using 10 μl QuantiFast SYBR Green PCR master mix, 4 μl of cDNA template with 1 μM of each primer in a total reaction volume of 20 μl. The PCR was run for 40 cycles and the thermal cycling conditions were as follows: initial heat activation at 95°C for 10 min; denaturation for 10 s at 95°C; combined primer annealing and extension for 60 s at 60°C. The fluorescence signal was measured at the end of each extension step. Fluorescence emission readings were analyzed using Rotor-Gene Q software (Qiagen, Germany). The data were presented as the relative gene expression (%) of the target gene expression, normalized to the housekeeping gene GAPDH, compared to the non-treated group.

### Statistical analysis

All results were expressed as mean ± standard deviation (*SD*) of at least three independent experiments using Student's *t*-test. *P* < 0.05 were considered significant.

## Author contributions

JT performed all the *in vitro* experiments in this study. JP provided advice and helped in the cell-related experiments. LK supervised and advised on the experiments. LS, JZ, and CW performed the qualitative and quantitative analysis of YSC in this study.

### Conflict of interest statement

The authors declare that the research was conducted in the absence of any commercial or financial relationships that could be construed as a potential conflict of interest.
